# Nanoscale Relationship Between CD4 and CD25 of T Cells Visualized with NSOM/QD-Based Dual-Color Imaging System

**DOI:** 10.1186/s11671-015-1130-x

**Published:** 2015-10-26

**Authors:** Jinping Fan, Xiaoxu Lu, Shengde Liu, Liyun Zhong

**Affiliations:** Guangdong Provincial Laboratory of Nanophotonic Functional Materials and Devices, South China Normal University, Guangzhou, 510006 Guangdong China

**Keywords:** Near-field scanning optical microscopy, Quantum dots, T cells, TCR/CD3, CD4, CD25

## Abstract

In this study, by using of near-field scanning optical microscopy (NSOM)/immune-labeling quantum dot (QD)-based dual-color imaging system, we achieved the direct visualization of nanoscale profiles for distribution and organization of CD4 and CD25 molecules in T cells. A novel and interesting finding was that though CD25 clustering as nanodomains were observed on the surface of CD4^+^CD25^high^ regulatory T cells, these CD25 nanodomains were not co-localized with CD4 nanodomains. This result presented that the formation of these CD25 nanodomains on the surface of CD4^+^CD25^high^ T cells were not associated with the response of T cell receptor (TCR)/CD3-dependent signal transduction. In contrast, on the surface of CD4^+^CD25^low^ T cells, CD25 molecules distributed randomly without forming nanodomains while CD4 clustering as nanodomains can be observed; on the surface of CD8^+^CD25^+^ T cells, CD25 clustering as nanodomains and co-localization with CD8 nanodomains were observed. Collectively, above these results exhibited that TCR/CD3-based microdomains were indeed required for TCR/CD3-mediated T cells activation and enhanced the immune activity of CD4^+^CD25^low^ T cells or CD8^+^CD25^+^ T cells. In particular, it was found that the formation of CD25 nanodomains and their segregation from TCR/CD3 microdomains were the intrinsic capability of CD4^+^CD25^high^ T cells, suggesting this specific imaging feature of CD25 should be greatly associated with the regulatory activity of CD4^+^CD25^high^ T cells. Importantly, this novel NSOM/QD-based dual-color imaging system will provide a useful tool for the research of distribution-function relationship of cell-surface molecules.

## Background

CD4^+^CD25^high^ regulatory T cells play an important role in suppression of a wide range of immune responses targeting various microbes, intracellular parasites, allergens, allo-antigens, and tumors [1–6]. Specially, it has been demonstrated that the effector function and regulatory activity of CD4^+^CD25^high^ T cells depend at least partially on T cell receptor (TCR)-major histocompatibility complex (MHC) interactions [7]. A lot of evidences exhibit that TCR/CD3 microdomains are a precondition to induce the immunologic synapse, in which a variety of signaling molecules are recruited to TCR/CD3 microdomains and facilitate T cell activation [8–11]. In recent years, combing dipole-polarization and dual-color imaging, we have constructed a near-field scanning optical microscopy (NSOM) and quantum dot (QD)-based nanosystem to visualize nanoscale distribution and organization of antigen-specific TCR/CD3, co-receptor CD4, CD8, as well as the nanospatial relationship between TCR/CD3 and CD4 or CD8 in a sustained T cells activation. The obtained results show that CD4 or CD8 are clustered and co-localized with TCR/CD3 microdomains in a sustained T cells activation, and the nanoscale imaging information supplies the convincing evidence in the molecular mechanism study of T cell activation signaling [12–14]. Up to now, it remains controversial about the molecular mechanism of CD4^+^CD25^high^ regulatory T cells-mediated suppression of autoimmunity [15, 16]. Moreover, there is no nanoscale information conceiving whether the distribution and organization of CD25 on the surface of CD4^+^CD25^high^ T cells are associated with its regulatory activity, the direct molecule imaging of nanoscale relationship between CD25 and CD4 has not been reported.

In this study, by using our homemade NSOM/QD-based dual-color imaging system, we intend to directly visualize nanoscale nanospatial relationship between CD4 and CD25 of CD4^+^CD25^high^ regulatory T cells, further detect whether the distribution and organization of CD25 on the surface of CD4^+^CD25^high^ T cells are associated with its regulatory activity.

## Methods

### Samples

We obtained human peripheral blood from the Blood Bank at University of Illinois at Chicago Hospital with formal agreement by the healthy volunteers, with the approval of the regional ethics committee. Peripheral blood mononuclear cells (PBMC) were isolated by Ficoll-Hypaque gradient centrifugation and washed with phosphate-buffered saline (PBS) as described by our previous reports [12].

### Antibodies and Reagents

We purchased anti-CD3-coated 96-well plates from BD Biosciences. Roswell Park Memorial Institute (RPMI)-1640 culture medium was obtained from GibcoBRL Corp. Rabbit anti-human CD4 and CD8 were purchased from Dako. Biotinylated anti-human CD25 was obtained from Sigma. The following antibodies and QDs were purchased from Invitrogen: biotinylated anti-mouse IgG, anti-rabbit IgG (H + L)-conjugated QD 655, and streptavidin-conjugated QD 605. Additionally, before the antibody labeling, all QDs were centrifuged to remove aggregates of QDs.

### T Cell Stimulation and Immune Staining

To achieve T cells co-stimulation with anti-CD3/anti-CD28 antibodies (Abs), we first seeded PBMC at a cell density of 2 × 10^5^ cells/mL onto anti-CD3 antibody (Ab)-coated 96-well plates, and then co-cultured them with 5 ng/mL anti-CD28 Ab for 12 and 48 h, respectively, in Roswell Park Memorial Institute (RPMI) 1640, containing 10 % FBS at 37 °C in a 5 % CO_2_ atmosphere.

To rule out the possibility of non-specific activation of T cells induced by antibody labeling in T cell immunostaining, first of all, we chose 2 % formalin/PBS solution to fix T cells. In the first color labeling, CD25 molecules were labeled with biotinylated anti-human CD25 antibody and then with streptavidin-conjugated QD 605. In the second color labeling, CD4 (or CD8) were labeled with rabbit anti-human CD4 (or CD8) and then with anti-rabbit IgG (H + L)-conjugated QD 655. Finally, the cells were further fixed through using 2 % formalin/PBS solution. Meanwhile, to remove any unbound antibody or QDs in above each labeling step, the cells were washed twice through employing FBS/PBS, in which the size of Ab or streptavidin-conjugated QD was about 25 nm, and the size of CD4 or CD8 or CD25 was about 1–5 nm, respectively. Following, to spread double distilled water suspensions of cells onto glass cover slides effectively, all glass cover slides were pretreated with poly-l-lysine (Sigma) and air dried at room temperature performed in our NSOM imaging study. Furthermore, as described in our previous reports [12], to avoid the possibility of non-specific labeling, the control labeling was also performed with isotype control antibody simultaneously.

In addition, to evaluate T cells activity and its regulatory activity, we respectively detected two cytokine productions of IL-2 and IL-10 in supernatant by ELISA in accordance with the manufacturer’s instruction (Bender Medsystems, Austria), in which CD4^+^CD25^+^ T cells and CD8^+^CD25^+^ T cells were purified with magnetic affinity cell sorting (MACS) approach though using the manufacturer’s instruction (Miltenyi Biotec, Sunnyvale, CA, USA).

### NSOM/QD-Based Dual-Color Imaging System

**Fig. 1:**
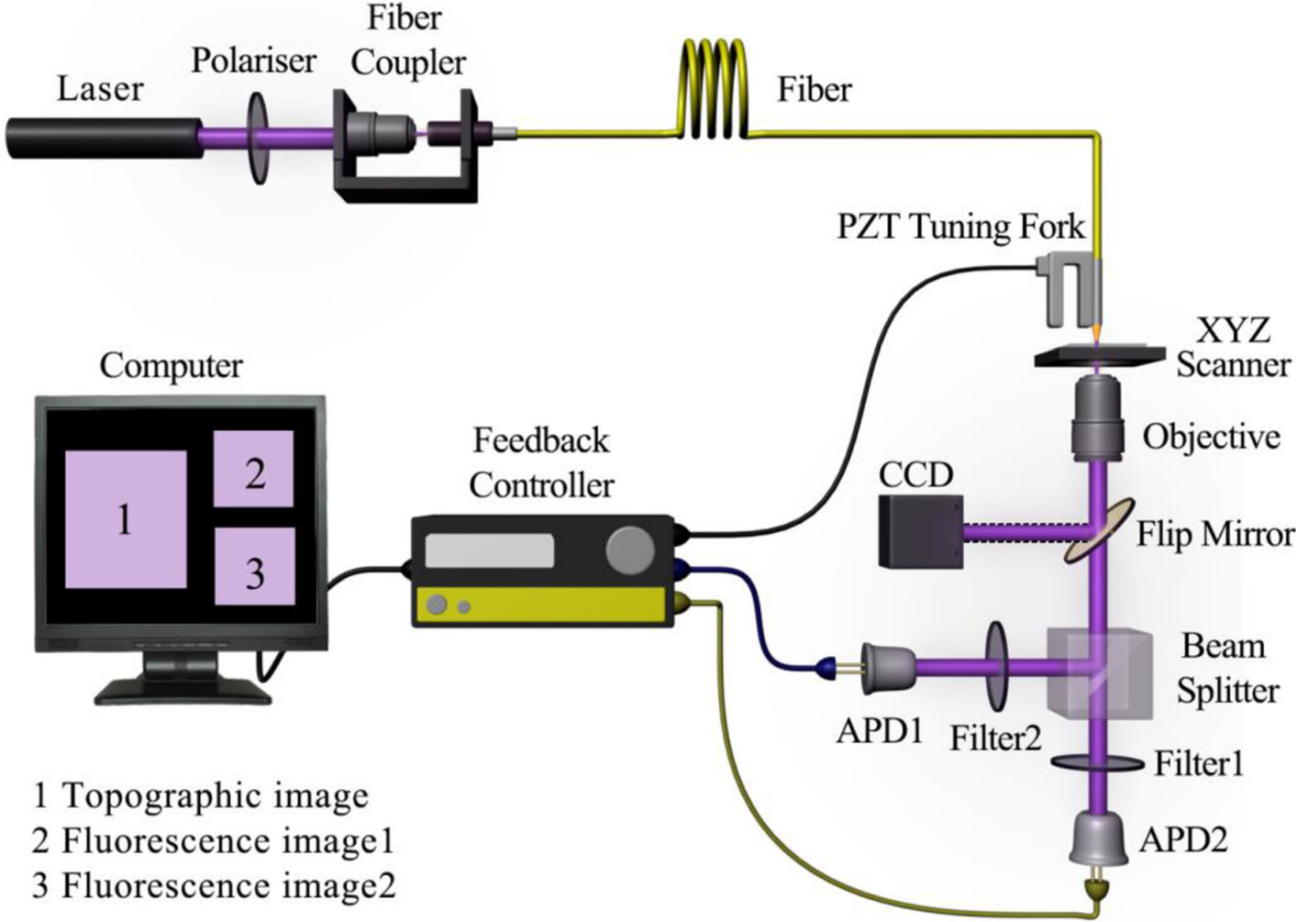
Schematic of the NSOM/QD-based dual-color imaging system, in which the charge-coupled device (CCD) was used as the monitor of optical fiber probe adjustment, and two avalanche photodiodes (APD) and two filters (605 ± 10 or 655 ± 10 nm) were employed as the detectors of two color fluorescence images, respectively

NSOM system can simultaneously obtain the topographic and optical data. In this work, an Aurora-3 NSOM system (Veeco, Santa Barbara, CA, USA) was employed to perform nanoscale imaging. The schematic layout of the NSOM/QD-based dual-color imaging system was shown in Fig. 1. A continuous wave semiconductor laser with wavelength of 404 nm and excitation intensity of 120 W/cm^2^ (Coherent, USA) was utilized as excitation source and launched into a single mode optical fiber (Thorlabs Inc, USA), while the other side of optical fiber was connected to an aluminum-coated probe with an aperture diameter of 50–80 nm (Veeco, USA). The probe tip was mounted onto a piezoelectric quartz tuning fork with the resonance frequency about 93 kHz, by tuning fork-based shear-force feedback, the probe-sample distance was maintained at a constant of 10 nm. Note that the previous study has demonstrated that no significant difference appeared in full width at the half maximum (FWHM) of fluorescent spots while different probes were used. In our homemade NSOM dual-color system, to achieve two color fluorescence information, the transmission light collected with a ×40, NA 0.65 objective (Olympus, Japan) was split into two beams though employing a cube beam splitter (Newport Inc., USA), and passed through two optical filters of 655 ± 10 and 605 ± 10 nm (Newport Inc., USA), which were used to separate the fluorescence from the excitation light and the background, and then detected by two APDs (PerkinElmer, Canada) in 0° and 90°, respectively. In addition, a XY stage with full scanning range of 30 × 30 μm was used to fix the measured sample, in which a video camera was employed to search for the regions of interest. The measured size of image was 400 × 400 scanning lines and the integration time was 30 ms, in which most of the captured images were performed a slight low-pass filtering before data processing. Our previous study has demonstrated that all above parameters were stable and reproducible during the repeated scanning [12].

### Image Processing and Data Analyses

To achieve high-quality NSOM fluorescence image and topographic image, firstly, we performed preprocessing for all captured images with leveling and the convolution by SPMLab 6.02 software (Veeco, USA), in which each pixel gray level was processed by its neighborhood averaging to reduce the influence of the stray noise points, so the image information lost will be very small. Subsequently, we chose Matlab 7.0 to perform the following image processing and analyses. (1) The color-coded technique in red and blue were implemented on the two fluorescence images captured by NSOM simultaneously, showing two labeled molecules (CD4 or CD8, as well as CD25); (2) a two-dimensional merged image of two color fluorescence images was obtained through using the intensity superposition algorithm of point to point (pixel to pixel), which expressed the intensity sum of pixel to pixel for one color fluorescence image to another color fluorescence image; (3) The fluorescence intensity and FWHM distribution of fluorescent spots, the fluorescence intensity of single QD, and the number of QD molecules in each fluorescence spot were calculated as following: since it has been found that a typical count rate for individual QD 655 and QD 605 were about 7000 and 4500 counts/s, respectively, at the excitation laser intensity of 120 W/μm^2^, and these values were reproducible in repeat experiments [12]. Moreover, one-on-one interaction between the conjugated QD and antigen or biotin was the favor in a saturation binding condition. Therefore, the QD numbers to correlate the molecule numbers were calculated on the basis of the conservative assumption that the QD: secondary Ab: primary Ab: target molecule = 1:1:1:1, in which the intensity of each spot was determined by adding all photon counts with a contour of 15 % of the peak intensity; (4) Using the average fluorescence intensity to indicate the average QD numbers, we can calculate the molecular density as dividing the molecule numbers over the nanodomains areas; (5) All data were expressed as mean ± standard error of the mean (SEM), Student’s *t* test for the comparison of means was employed to show the statistical difference of molecular density, as well as the percentages of molecules that localized into nanodomains of different groups, and *P* value less than 0.05 is considered to be statistically significant.

## Results

### Most of CD25 Were Clustered as Nanodomains but Not Co-localized with CD4 Nanodomains in CD4^+^CD25^high^ T Cells

Several research groups have demonstrated that one specific imaging feature for TCR/CD3-mediated T cells activation is TCR/CD3 clustering at the center of the T cell/antigen presentation cell (APC) interface [9, 16], and CD4 function as co-receptor stabilizing MHCp-TCR/CD3 interaction for enhancing TCR/CD3 signaling. Moreover, our recent work also showed that co-receptor CD4 molecules were clustered as nanodomains and co-localized with TCR/CD3 microdomains in a sustained T cells activation [12]. For convenience, in this study, we took advantage CD4 clustering as readout of TCR/CD3 signaling. In addition, since IL-2 production is required for TCR/CD3-mediated T cells activation [17], we choose IL-2 production as the activity parameter of TCR/CD3-mediated T cells activation.

CD4^+^CD25^high^ regulatory T cells, revealing the lack of the response upon TCR/CD3-mediated activation, are greatly different from conventional CD4^+^CD25^low^ T cells with the positive immune response. Therefore, we ask whether CD25 nanoscale imaging of CD4^+^CD25^high^ regulatory T cells is different from that of CD4^+^CD25^low^ T cells. To solve this issue, we employed our homemade NSOM/QD-based dual-color imaging system to detect the nanoscale spatial relationship between CD4 and CD25 for different subset of T cells. Furthermore, since IL-10 production is an important immunoregulatory cytokine and the effect of CD4^+^CD25^high^ regulatory T cells is mainly mediated by IL-10 [18]; thus, we choose IL-10 production as the evaluation parameter of CD4^+^CD25^high^ T cells regulatory activity.

**Fig. 2:**
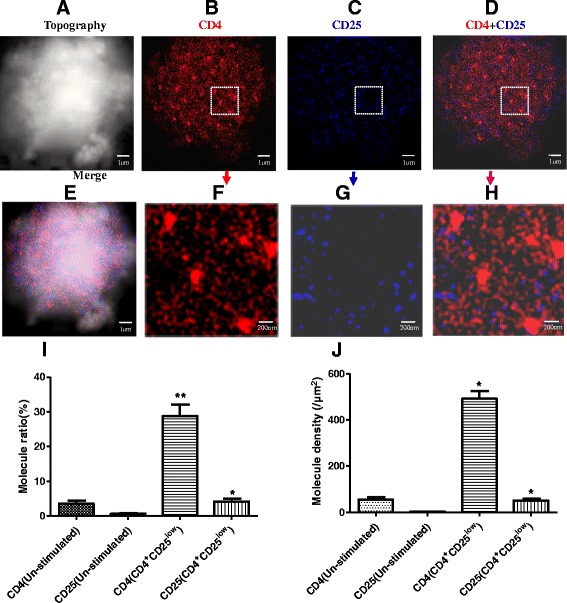
Simultaneous nanoscale dual-color imaging of CD4 and CD25 on the surface of CD4^+^CD25^low^ T cells through using NSOM/QD system. **a** T cell topography. **b** Fluorescence image of CD4 labeled with QD-655 (*red*). **c** Fluorescence image of CD25 labeled with QD-605 (*blue*). **d** Merge of CD4 and CD25 two color fluorescence images. **e** Merge of cell topography and two color fluorescence images. **f**–**h** Zoom images of the areas as indicated by the *squares* on (**b**–**d**), respectively. **i** The percentage numbers of CD4 or CD25 molecules arrayed to form nanodomains. **j** Molecule density of CD4 or CD 25 nanodomains. Data were expressed as mean ± SEM in (**i**, **j**), *P < 0.02, **P < 0.01 (**i**) and *P < 0.01 (**j**) compared with control

**Fig. 3:**
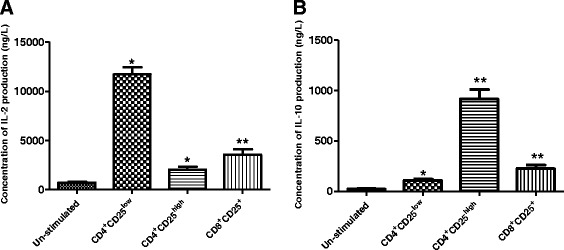
**a** IL-2 concentration of different subset T cells. **b** IL-10 concentration of different subset T cells, in which T cells were co-stimulated with anti-CD3/anti-CD28 Abs. Data were expressed as mean ± SEM in (**a**, **b**), *P < 0.001, **P < 0.0005 (**a**) and *P < 0.002, **P < 0.001 (**b**) compared with control

To discriminate CD4^+^CD25^low^ T cells and CD4^+^CD25^high^ T cells during T cells activation, we defined these two subsets of CD4^+^ T cells with different CD25 expression based on the anti-CD3/anti-CD28 Abs co-stimulated time: (i) CD4^+^CD25^low^ T cells, stimulated time is less than 12 h; (ii) CD4^+^CD25^high^ T cells, stimulated time is more than 48 h. Furthermore, to evaluate the nanoscale distribution and organization of CD4 and CD25, we defined nanostructures of these molecules as nanoclusters with size of 100–200 nm and nanodomains with size of 200–500 nm, respectively. Additionally, our recent work showed that most of the CD4 molecules on the resting T cell were detected as 70–140 nm nanoclusters and distinct with 40–50-nm distance from each other under the high resolution NSOM [12], but CD25 is expressed on the activated T cells. As an initial effort, to directly visualize CD4 interaction with CD25 during T cells activation through using NSOM-QD-based dual-color system, we first imaged nanoscale distribution and relationship of CD4 and CD25 on the cell surface of CD4^+^CD25^low^ T cells with the anti-CD3/anti-CD28 Abs co-stimulation. Interestingly, on the surface of CD4^+^CD25^low^ T cells, about 30 % of CD4 molecules clustering as nanodomains of 200–350 nm can be observed, but no visible CD25 clustering were observed and nearly all of the CD25 molecules distributed randomly on the cell surface (Fig. 2a–c). As described in our recent work [12], in CD4^+^CD25^low^ T cells, these CD4 nanodomains were interacted and co-localized with TCR/CD3 microdomains. Accordingly, as shown in Fig. 3a, the concentration of IL-2 production of CD4^+^CD25^low^ T cells (11700.4 ± 872.3 ng/L) was nearly 17 times bigger than that of un-stimulated T cells (704.8 ± 37.6 ng/L), indicating that TCR/CD3-based microdomains were indeed required for TCR/CD3-mediated T cells activation and enhanced the immune activity of CD4^+^CD25^low^ T cells.

**Fig. 4:**
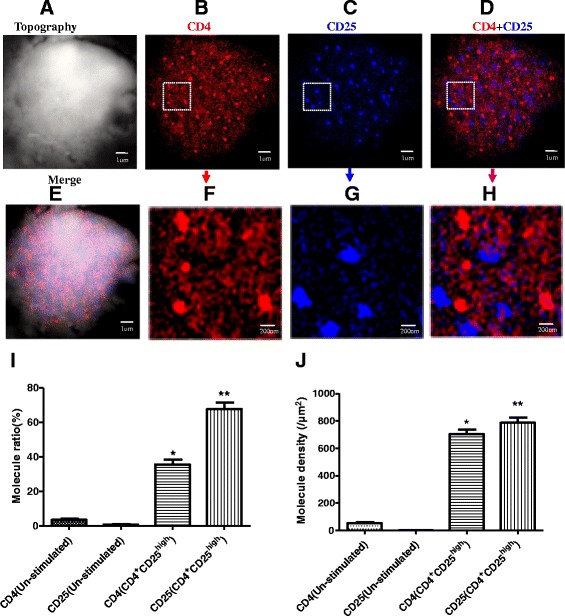
Simultaneous nanoscale dual-color imaging of CD4 and CD25 on the surface of CD4^+^CD25^high^ T cells through using NSOM/QD system. **a** T cell topography. **b** Fluorescence image of CD4 labeled with QD-655 (*red*). **c** Fluorescence image of CD25 labeled with QD-605 (*blue*). **d** Merge of CD4 and CD25 two color fluorescence images. **e** Merge of cell topography and two color fluorescence images. **f**–**h** Zoom images of the areas as indicated by the *squares* on (**b**–**d**), respectively. **i** The percentage numbers of CD4 or CD25 molecules arrayed to form nanodomains. **j** Molecule density of CD4 or CD 25 in nanodomains. Data were expressed as mean ± SEM in (**i**, **j**), *P < 0.03, **P < 0.02 (**i**) and *P < 0.01, **P < 0.005 (**j**) compared with control

For CD4^+^CD25^high^ T cells, using NSOM-QD-based dual-color imaging system, though a lot of CD4 clustering and CD25 clustering with the size of 200–350 nm also can be observed on the cell surface (Fig. 4a–c, e–g), but surprisingly, these specific CD25 nanodomains were not co-localized with CD4 nanodomains (Fig. 4d, h). The statistical analysis showed that though the average molecule density of CD25 in nanodomains (789 ± 45/μm^2^) was nearly the same with that of CD4 in nanodomains (705 ± 34/μm^2^) (Fig. 4j), but the percentage of CD25 molecules that arrayed to form nanodomains (67.7 ± 3.4 %) was nearly two times bigger than that of CD4 molecules that arrayed to form nanodomains (35.7 ± 2.2 %) (Fig. 4i). Recently, it has been reported that CD4 clustering was greatly associated with TCR/CD3-mediated signaling transduction, in which CD4 clustering and its co-localization with TCR/CD3 microdomains will lead TCR-mediated signaling amplifies and facilitate T cells activation. In response, as shown in Fig. 3a, b, the concentration of IL-2 production of CD4^+^CD25^high^ T cells (2054.8 ± 264.7 ng/L) was about 80 % lower than that of CD4^+^CD25^low^ T cells (11700.4 ± 872.3 ng/L) while the concentration of IL-10 production in CD4^+^CD25^high^ T cells (918.4 ± 86.7 ng/L) was nearly 8.5 times bigger than that of CD4^+^CD25^low^ T cells (109.8 ± 8.6 ng/L). Obviously, these results exhibited that the formation of CD25 nanodomains was not associated with TCR/CD3-mediated signaling transduction, but such a large number of CD25 clustering would implicate a positive feedback to facilitate the regulatory activity of CD4^+^CD25^high^ T cells, suggesting that this specific imaging feature of CD25 was possibly associated with the regulatory activity and the effector function of CD4^+^CD25^high^ T cells.

### Most of CD25 Were Clustered as Nanodomains and Co-localized with CD8 Nanodomains in CD8^+^CD25^+^ T Cells

To determine whether the formation of CD25 nanodomains and its segregation from TCR/CD3 microdomains was the intrinsic event and specific capability of CD4^+^CD25^high^ T cells, we also utilized NSOM/QD-based dual-color imaging system to visualize the nanospatial relationship between CD8 and CD25 in CD8^+^CD25^+^ T cells. As we know, like co-receptor CD4 in CD4^+^CD25^+^ T cells, it was also well accepted that CD8 function as co-receptors stabilizing MHCp-TCR/CD3 interaction for promoting TCR/CD3 signaling in CD8^+^CD25^+^ T cells. Now, collecting evidence indicates that CD8^+^CD25^+^ T cells also reveal a little positive immune response upon TCR/CD3-mediated activation [19]. Moreover, our recent work also showed that co-receptor CD8 were clustered as nanodomains and co-localized with TCR/CD3 microdomains during CD8^+^CD25^+^ T cells activation. Similarly, we also took advantage CD8 clustering as readout of TCR/CD3 signaling in the following research.

**Fig. 5:**
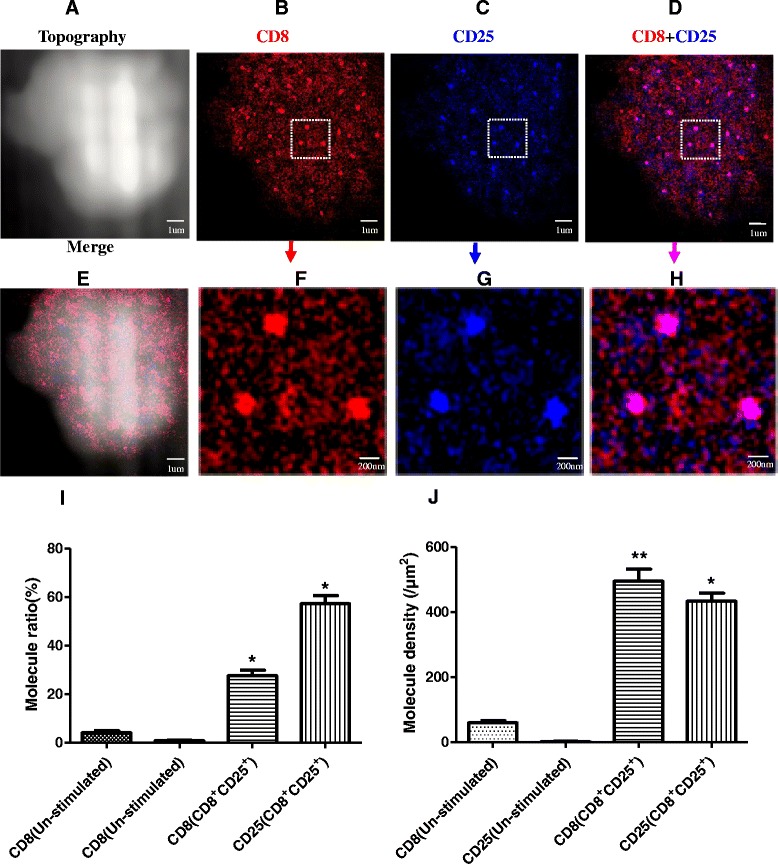
Simultaneous nanoscale dual-color imaging of CD4 and CD25 on the surface of CD8^+^CD25^+^ T cells though using NSOM/QD system. **a** T cell topography. **b** Fluorescence image of CD8 labeled with QD-655 (*red*). **c** Fluorescence image of CD25 labeled with QD-605 (*blue*). **d** Merge of CD8 and CD25 two color fluorescence images. **e** Merge of cell topography and two color fluorescence images. **f**–**h** Zoom images of the areas as indicated by the *squares* on (**b**–**d**), respectively. **i** The percentage numbers of CD8 or CD25 molecules arrayed to form nanodomains. **j** Molecule density of CD8 or CD25 nanodomains. Data were expressed as mean ± SEM in (**i**, **j**), *P < 0.02, **P < 0.01 (**i**) and *P < 0.02, **P < 0.005 (**j**) compared with control

Interestingly, after anti-CD3/anti-CD28 Abs co-stimulation of 48 h, both CD8 clustering and CD25 clustering as nanodomains of 200–300 nm can be observed on the surface of CD8^+^CD25^+^ T cells through using NSOM-QD-based dual-color system (Fig. 5a–c), moreover, all of CD25 nanodomains were co-localized with CD8 nanodomains. The statistical analysis showed that though the average molecule density of CD25 in nanodomains (434 ± 29/μm^2^) was nearly the same with CD8 in nanodomains (495 ± 41/μm^2^) (Fig. 5j), but the percentage of CD25 molecules that arrayed to form nanodomains (57.2 ± 4.1 %) was about two times bigger than that of CD8 molecules (27.6 ± 2.7 %) (Fig. 4i). Accordingly, as shown in Fig. 3a, b, the concentration of IL-2 production of CD8^+^CD25^+^ T cells (3579.2 ± 423.5 ng/L) was nearly 75 % higher than that of CD4^+^CD25^high^ T cells (2054.8 ± 264.7 ng/L) while the concentration of IL-10 production of CD8^+^CD25^+^ T cells (228.6 ± 27.3 ng/L) was about 75 % lower than that of CD4^+^CD25^high^ T cells (918.4 ± 86.7 ng/L). These results possibly exhibited that the formation of CD25 nanodomains and their co-localization with CD8 nanodomains were associated with TCR/CD3-mediated signaling, and this nanoscale imaging feature would play an important role to enhance the immune activity CD8^+^CD25^+^ T cells.

In summary, the above results provided evidence demonstrating that TCR/CD3-based microdomains were indeed required for TCR/CD3-mediated T cells activation. Moreover, it was exhibited that the formation of TCR/CD3-based microdomains will play an important role in enhancing the immune activity of CD4^+^CD25^low^ T cells or CD8^+^CD25^+^ T cells. Specially, it was found that the formation of CD25 nanodomains and their segregation from TCR/CD3 microdomains were the intrinsic capability of CD4^+^CD25^high^ T cells, suggesting that this specific imaging feature of CD25 should be greatly associated with the regulatory activity of CD4^+^CD25^high^ T cells.

## Discussion and Conclusions

Combing the immunofluorescence labeling approach and NSOM/QD-based nanoscale imaging technique, several groups and our previous study have demonstrated that TCR/CD3 microdomains are required for the full T cells activation, in which a variety of signaling molecules including co-receptor CD4 or CD8 are recruited to TCR/CD3 microdomains and thus facilitate T cells activation [12, 14, 20–22]. As we know, CD4^+^CD25^high^ T cells reveal a specific regulatory activity [13, 14], which is different from the conventional CD4^+^CD25^low^ T cells with the positive immune response. We therefore ask what is the difference of the nanoscale profiles for distribution and organization of CD4 and CD25 between CD4^+^CD25^high^ T cells and CD4^+^CD25^low^ T cells. Up to now, whether CD25 are clustered and co-localized with CD4 clustering in CD4^+^CD25^+^ T cells keep unknown and the direct molecule imaging of nanoscale relationship between CD25 and CD4 has not been reported.

In the current study, by using our homemade NSOM/QD-based dual-color imaging system, we achieved the direct visualization of the nanoscale profiles for distribution and organization of CD4 and CD25 in CD4^+^CD25^high^ regulatory T cells or CD4^+^CD25^low^ T cells. Moreover, based on the detection of IL-2 production and IL-10 production in different subset of T cells, some information about the relationship between the distribution and organization of CD25 and their effector function were presented. Interestingly, after anti-CD3/anti-CD28 Abs co-stimulation, like CD4 clustering as nanodomains, CD25 clustering as nanodomains were also observed on the surface of CD4^+^CD25^high^ T cells, but these specific CD25 nanodomains were not co-localized with CD4 nanodomains. However, in CD4^+^CD25^low^ T cells, CD25 distribute randomly without forming nanodomains while CD4 clustering as nanodomains were observed on the cell surface. Accordingly, the concentration of IL-2 production of CD4^+^CD25^high^ T cells was greatly lower than that of CD4^+^CD25^low^ T cells, but the concentration of IL-10 production of CD4^+^CD25^high^ T cells was greatly higher than that of CD4^+^CD25^low^ T cells. These results possibly exhibited that the formation of CD25 nanodomains was associated with the regulatory activity of CD4^+^CD25^high^ T cells but did not dependent on TCR/CD3-mediated signaling.

In addition, in CD8^+^CD25^+^ T cells, both CD8 clustering and CD25 clustering as nanodomains were observed on the cell surface, moreover these CD25 nanodomains were co-localized with CD8 nanodomains, showing that the formation of CD25 nanodomains and their segregation from TCR/CD3 microdomains was the intrinsic imaging feature of CD4^+^CD25^high^ regulatory T cells. That is to say, this specific imaging feature of CD25 on CD4^+^CD25^high^ T cells should be associated with its regulatory activity; and the difference of nanoscale profiles for distribution and organization of CD25 between CD4^+^CD25^high^ T cells and CD4^+^CD25^low^ T cells should be closely related to their individual effector function. Importantly, by using this novel NSOM/QD-based dual-color imaging system, we can achieve the direct visualization of the nanoscale nanospatial relationship between cell-surface molecules, and this will provide more useful information for the research of distribution-function relationship of cell-surface molecules.
